# Frameworks for assessing digital health technologies: a scoping review

**DOI:** 10.1017/S0266462326103523

**Published:** 2026-03-09

**Authors:** Hendrikje Rödiger, Laura Franziska Marie Wittich, Reinhard Jeindl, Yui Hidaka, Dionne Bowie, Juan Carlos Rejón-Parrilla, Zoe Garrett, Reinhard Busse, Cornelia Henschke

**Affiliations:** 1 Department of Health Care Management, Technische Universität Berlin, Germany; 2 Austrian Institute for Health Technology Assessment GmbH, Austria; 3 National Institute for Health and Care Excellence, UK; 4 Health Technology Assessment Area (AETSA), Andalusian Public Foundation Progress and Health (FPS), Seville, Spain; 5 Institute of General Practice and Interprofessional Care, University Hospital Tübingen, Germany

**Keywords:** health technology assessment, digital health, assessment frameworks, evidence requirements, lifecycle approach, scoping review

## Abstract

**Objectives:**

The rapid evolution of digital health technologies (DHTs) presents distinct challenges for health technology assessment (HTA). Existing HTA frameworks, largely designed for conventional health interventions, may not sufficiently address these unique complexities. This scoping review provides an overview of existing assessment frameworks for DHTs, analyzing their purpose and the guidance they offer within the domains of the EUnetHTA Core Model.

**Methods:**

The review followed the Joanna Briggs Institute methodology and PRISMA-ScR guidelines. The literature was identified through searches in PubMed and Embase, covering publications from 2015 to 2024 in English or German, and was complemented by a manual hand search. The studies were screened and analyzed using Covidence, with data categorized inductively based on the EUnetHTA Core Model domains.

**Results:**

Of 3,576 screened records, 15 met inclusion criteria; an additional 45 frameworks were identified through hand searching, resulting in a total of 60 frameworks. Most frameworks focused on digital health applications (68 percent), while only a few addressed technologies such as artificial intelligence (2 percent). The frameworks primarily provide guidance on assessment, with varying focus on evidence requirements. The domains of the EUnetHTA Core Model were variably represented across the frameworks. Technical characteristics were most frequently addressed, while ethical, legal, and organizational domains received limited attention.

**Conclusions:**

This review highlights the diversity of existing frameworks for DHT assessment. This emphasizes the potential relevance of a future standardized framework that contains explanations of the methodological approach to the assessment of DHTs and is modularly customizable depending on the type of technology.

## Introduction

Given the challenges of a changing burden of disease, demographic transition, rising health expenditures, and concerns about the quality and value of many interventions (1–3), the urgency to transform healthcare systems is growing. The rapid development of digital health technologies (DHTs) is associated with the expectation of improving accessibility, affordability, and quality of care (4). As with other technologies, the value of DHTs must be evaluated in a multidisciplinary, systematic process across their life cycle to inform appropriate uptake (5;6). This process is known internationally as health technology assessment (HTA). It informs decision-making to promote equitable, efficient, and high-quality healthcare systems by assessing domains such as clinical effectiveness, safety, costs, and broader social, ethical, and organizational impacts (5).

However, DHTs may differ from traditional healthcare technologies in their rapid development, unique technological features, and complexities of integration into routine care (7). In response, a variety of frameworks have been developed, serving purposes ranging from regulatory guidance to coverage and reimbursement within public health systems. Yet, despite these developments, many generic or specialized assessment frameworks continue to fall short of capturing the distinctive challenges posed by DHTs (8). For instance, the widely used Core Model of the European Network for HTA (EUnetHTA) outlines domains and methodological approaches for HTA but has limitations when applied to DHTs, given their distinctive characteristics and rapid evolution (9;10). To adequately address the rapid technological updates and continuous evolution of DHTs, assessment frameworks may need to adopt a life cycle-oriented perspective that allows for ongoing evaluation and adaptation over time (6). As Moshi et al. emphasized, many frameworks for mobile health applications lack coverage of key HTA domains, rendering them unsuitable for comprehensive evaluation (11). In addition, existing classifications often struggle to keep pace with the speed and complexity of DHT innovations (12). A systematic literature review investigating published assessment guidelines for digital health interventions found no existing value assessment framework capable of evaluating their multidimensional outcomes across diverse stakeholder perspectives (13).

In light of these limitations, current HTA frameworks may not be sufficient for assessing specific DHTs due to their distinct benefit and risk profiles (14). Evaluating DHTs may require addressing unique challenges, such as generating appropriate evidence for a wide set of assessment items, understanding how digital technologies interact with users, the dependence of effectiveness on these interactions, organizational and technical implications including system integration, diagnostic potential, impact on health outcomes, and determining fair pricing (15).

To address these challenges, robust DHT evaluation frameworks should encompass clinical effectiveness and safety as they are fundamental aspects of any health intervention, including DHTs. Frameworks should therefore incorporate rigorous methodologies for assessing the impact of DHTs on health outcomes, including both benefits and potential harms (16). In addition, technical aspects, particularly security and privacy measures, must be thoroughly addressed due to the sensitive nature of health data (17). Furthermore, frameworks may incorporate a checklist on interoperability, ensuring that DHTs are capable of securely exchanging data with other systems, which is crucial for seamless integration into healthcare infrastructures (18;19).

Frameworks should also include methods for assessing the costs and benefits of DHTs compared to alternative interventions, as cost-effectiveness remains a critical factor in healthcare decision-making (20). Patient and social aspects, such as user experience, are becoming increasingly important, as DHTs must be user-friendly and accessible to ensure adoption and effective use (21–24).

DHTs also raise a range of ethical considerations, including potential impacts on health equity, making it essential for frameworks to incorporate mechanisms to assess and mitigate these risks (25;26). A holistic framework should not only reflect the traditional HTA domains but also explicitly integrate ELSI domains (ethical, legal, and social implications).

Several previous reviews have pursued similar objectives (8;11;27–30); however, this scoping review provides a more up-to-date synthesis and evaluates the extent to which existing frameworks address the assessment of DHTs throughout their life cycle, including their classification methodologies. It identifies both established and emerging value frameworks, analyzing their purpose and guidance within the domains of the EUnetHTA Core Model. In contrast to earlier reviews, this analysis applies a structured, domain-based synthesis that enables a systematic comparison of how existing frameworks address key assessment dimensions of DHT evaluation and incorporates a life cycle-oriented perspective to examine the extent to which life cycle-related aspects are reflected in the frameworks. This work is conducted as part of the ASSESS DHT project, funded by the European Union, which aims to consolidate existing methods and tools for DHT approval and to develop a new, generic framework for their assessment (31).

## Methods

### Overview of methods

A scoping review was conducted following the guidance provided by the Joanna Briggs Institute Reviewer Manual (32) and in accordance with the PRISMA-ScR (Preferred Reporting Items for Systematic Reviews and Meta-Analyses Extension for Scoping Reviews) reporting guidelines (33). A prospectively registered protocol was published at the Open Science Framework on 2 May 2024 (34). A literature search was performed in April 2024 using the PubMed and Embase databases.

This review aimed to identify, explore, and map available frameworks for assessing DHTs. The purpose and guidance provided by these frameworks were analyzed within the domains of the EUnetHTA Core Model. This comprises nine domains, which include 51 topics addressing 145 specific issues (9). In addition, the review examined whether these frameworks differentiate between various groups, classifications of DHTs or whether they included information on life cycle approach.

### Study inclusion and exclusion criteria

The inclusion and exclusion criteria were defined by employing the Joanna Briggs Institute PICo categories (Research questions in Population/Problem, Interest/Phenomenon of Interest, Context and Design) (32;35).

The review focused on frameworks addressing the assessment of DHTs (e.g., telemedicine, mobile health applications, or decision support systems based on artificial intelligence) that include at least one domain of the EUnetHTA Core Model. Frameworks exclusively targeting administrative or infrastructural digital systems, such as electronic medical records or hospital information systems, were not considered within the intended scope. Reviews, HTA methods guidance and policy documents, and qualitative and quantitative studies published between January 2015 and April 2024 were eligible for inclusion. Articles written in English and German were included.

Frameworks that did not focus on assessing DHTs were excluded. Frameworks in the following types of publications were excluded: study registers, protocols, conference abstracts, editorials, letters to the editor, commentaries, commemorative publications, errata, and dissertations.

### Search strategy

Searches of the literature were conducted for the period from January 2015 to April 2024 using two databases (PubMed and Embase) and defined search terms related to two core topics: digital health technologies and frameworks for their assessment, included in the title or abstract. The start date of January 2015 was chosen to capture the most recent developments in digital health assessment frameworks and to provide an updated synthesis compared to earlier reviews. The full search strategy is summarized in Supplementary File 1. The identification of relevant studies was complemented with a nonsystematic hand search and a snowballing search based on the references in the included studies, as well as citations of the included studies. The hand search included grey literature sources such as HTA agencies and networks known to publish methodological frameworks or guidance for digital health assessment. The list of member agencies from the International Network of Agencies for Health Technology Assessment was used to identify relevant institutions, and the websites of individual agencies were screened for frameworks and policy documents related to the assessment of digital health technologies. Relevant reviews (8;11;27–30) and frameworks of an analysis from the Evidence DEFINED project (36;37) were also examined, as they pursued similar objectives.

### Eligibility and data extraction

To manage the screening process and track agreement between reviewers, the Covidence tool (38) was used. Two reviewers (HR and LW) screened a random 20 percent sample of all unique records based on their titles and abstracts and discussed their results until a consensus on inclusion was reached. Agreement between them was sufficiently high when at least 80 percent raw agreement was reached so that the remaining records were screened by only one reviewer. Afterward, one reviewer (HR) screened all full-text articles included after title/abstract screening. A second reviewer (LW) screened all full-text articles excluded by the first reviewer during full-text screening.

An extraction sheet was developed, tested by two researchers, and following further adaptation created in the Covidence tool (38). The data extraction process was conducted by three researchers (HR, YH, and RJ), and any discrepancies were resolved by consensus.

Data on the following items were extracted: country, organization/HTA agency, author(s), year of publication, title, version of the framework, purpose of the assessment framework, groups/types/tiers of DHTs, scope of assessments (clinical, nonclinical, technological aspects), and evidence requirements for DHTs (e.g., choice of endpoints/outcomes, choice of comparator, study designs used for evidence generation), life cycle approach.

### Descriptive analysis of the data

Descriptive analyses were conducted on general framework characteristics (e.g., stated purpose), the grouping or classification of DHTs, and whether the framework targeted specific types of DHTs. In addition, data on evidence requirements and lifecycle stages were examined.

Framework content was analyzed using the domains of the EUnetHTA Core Model (see Table 1 for a full list of these domains) (9). First, the content of the individual frameworks (hereinafter referred to as “items”), including criteria, methods, and evidence requirements for assessing DHTs, was assigned to the nine HTA domains of the EUnetHTA Core Model. Subsequently, one researcher inductively developed categories for these items. This inductive approach also allowed for the identification of categories and items that extended beyond the predefined domains of the EUnetHTA Core Model. The results were then reviewed by at least one other researcher. Through iterative discussions among all researchers and by moving items back and forth, the items were refined and reallocated where necessary until consensus was achieved. The allocation of items to the EUnetHTA Core Model domains was guided by the content and context in which each item was described within the source framework. In some cases, the source frameworks did not include all EUnetHTA domains, and certain items could be classified under more than one domain, for example legal obligations under the ethical rather than the legal domain, or digital literacy under the organizational rather than the patients and social domain. In cases of overlap, items were assigned to the domain that best reflected their primary focus. Ultimately, the main categories, and in many cases, subcategories were established. A narrative synthesis was performed by summarizing the characteristics and results of the included studies in both table and text formats.

**Table 1.** Definitions and frequencies of the HTA domains in frameworks

HTA domain	Domain definition	Frequency in frameworks
Health Problem and Current Use (CUR)	Addresses target conditions, affected groups, epidemiology, technology use, burden on individuals and society, alternative treatment options, regulatory status, and usage requirements of the technology in question (9)	21 frameworks (35%)
Description and Technical Characteristics (TEC)	Provides details about the technology, including its development, purpose, users, usage, conditions addressed, and healthcare level (9)	45 frameworks (75%)
Safety (SAF)	Refers to any harmful effects resulting from the use of a health technology. Safety assessments are critical for benefiting patients and informing policies, although methods can vary based on diverse safety concerns across technologies (9)	27 frameworks (45%)
Clinical Effectiveness (EFF)	Evaluates whether a technology works under ideal conditions (efficacy) and in real-world healthcare settings (effectiveness). The focus is primarily on its practical application (9)	41 frameworks (70%),
Cost and Economic evaluation (ECO)	Informs value-for-money judgments by providing data on costs, and economic efficiency (9)	32 frameworks (53%)
Ethical Analysis (ETH)	Examines the social and moral norms and values relevant to the technology being assessed (9)	15 frameworks (25%)
Organisational Aspects (ORG)	Examines how resources (e.g., materials, skills, money, and attitudes) are organized for technology implementation and their impact on the organization and healthcare system (9)	11 frameworks (18%)
Patient and Social Aspects (SOC)	Focuses on patients or individuals receiving care through technology. Patient aspects address issues relevant to patients and caregivers, whereas social aspects concern specific groups of patients or individuals of interest in an HTA (9)	46 frameworks (77%)
Legal Aspects (LEG)	Intended to help HTA practitioners identify relevant rules and regulations, including those relating to patient rights, data protection and healthcare professionals (9)	14 frameworks (23%)

## Results

### Study selection and characteristics

**Figure 1.**
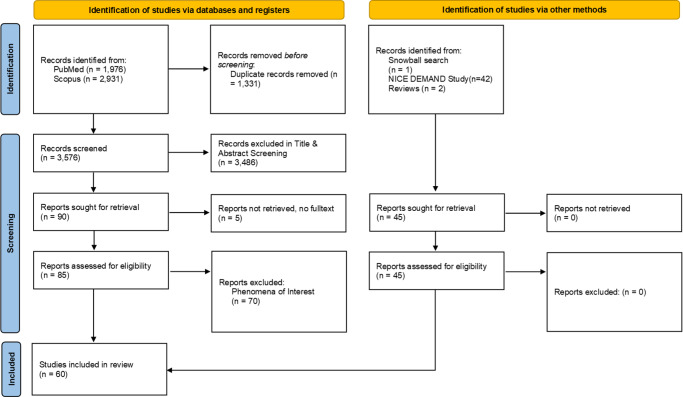
PRISMA flow chart illustrating the study identification and selection process.

After removal of duplicates, a total of 3,576 records were screened by title and abstract. Ninety articles were included in the full-text review. Of these, seventy-five articles were excluded, resulting in fifteen articles that were reviewed in full. The manual search included forty-five records, for example, through websites, systematic reviews, or frameworks included in the DEFINED study (36). In total, sixty articles were included in the synthesis (Figure 1). The list of included frameworks is provided in Supplementary File 2.

### General information of identified frameworks

The sixty identified frameworks for DHT assessment were published between 2015 and 2024. The individual purposes of the frameworks were summarized in themes. The predominant purpose theme of the frameworks was “Support & Guidance” (47 percent, providing recommendations for informed decisions on DHTs), followed by “Evaluation and Evaluation Standards” (28 percent, providing evaluation criteria) and “Quality Evaluation” (18 percent, focusing on quality assessment).

Only three frameworks explicitly classified DHTs into groups that informed differentiated assessment methods or evidence requirements. The NICE Evidence Standards Framework (ESF) (39) classifies DHTs based on their intended purpose, which allows them to be stratified into tiers based on the potential risk to service users and to the system. Segur-Ferrer et al. (10) adopted the ESF to structure the classification in their methodological framework. Lantzsch et al. (40) classify digital health applications according to four attributes: (i) application area, (ii) target group, (iii) function, and (iv) user, and define three levels of evidence requirements depending on the classification.

With regard to types of technologies, the majority of frameworks focused on “digital health applications” (68 percent) followed by “general digital health technologies” (not further defined) (20 percent), “telemedicine” (3 percent), “artificial intelligence” (AI) (2 percent), and “other” digital technologies (e.g., precision health innovations or sensor technologies) (7 percent).

Life cycle-related aspects were only rarely addressed in the identified frameworks, and few provided information on different assessment approaches during the life cycle of a DHT. Among the sixty frameworks analyzed, only ten provided information on life cycle stages, with seven of these mentioning life cycle stages in general terms (6;10;41–45) without any implications such as different assessment requirements. Notable exceptions included ongoing app evaluations throughout the entire development life cycle (17), early deployment standards for evidence-generation programs (39), and postmarket monitoring (46).

A greater number of frameworks (n = 24) addressed evidence requirements. Regarding the specific evidence required for DHTs, 21 percent outlined evaluation criteria, another 21 percent focused on testing and validation, while others covered aspects such as study designs (17 percent), specific evidence tiers or requirements (17 percent), availability of evidence (13 percent), and general levels of evidence (12 percent).

However, there is little information in the frameworks suggesting that the requirements vary depending on the type of DHT or its lifecycle stage. The NICE ESF (39;47) stands out as a comprehensive framework containing twenty-one standards (evaluation requirements). It also includes sixteen early deployment standards designed to support evidence generation programs, helping companies develop an evidence base for DHTs at an early deployment stage.

### Domains considered in identified frameworks

From the sixty frameworks, scientific articles, and guidelines identified, whether or not each framework addressed the domains of the EUnetHTA Core Model (9) was evaluated. The majority covered the “Patients and Social Aspects” (SOC) domain (77 percent), followed by “Description and Technical Characteristics” (TEC) (75 percent) and “Clinical Effectiveness” (70 percent) (EFF). Coverage of “Costs and Economic Evaluation” (ECO) (53 percent) and “Safety” (SAF) (45 percent) was moderate, while domains such as “Health Problem and Current Use” (CUR) (35 percent), “Ethical Analysis” (ETH) (25 percent), “Legal Aspects” (LEG) (23 percent), and “Organizational Aspects” (ORG) (18 percent) were addressed less frequently. Definitions of the domains and their frequency across frameworks are presented in Table 1. An overview of the domains covered by each framework is provided in Supplementary File 3.

**Figure 2.**
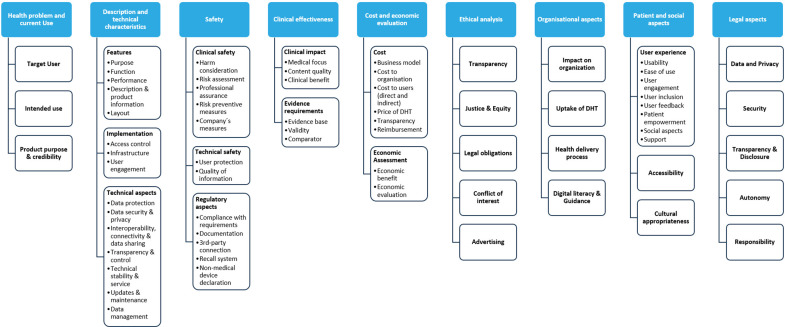
Main categories and subcategories assigned to the HTA domains.

The analysis included 660 items extracted from the frameworks. Out of all items, a categorization into main categories (with or without subcategories) per domain was established. A total of thirty-one main categories and forty-nine subcategories were created. The items were then assigned to the categories accordingly. Figure 2 illustrates the developed main categories and subcategories across the HTA domains.

**Figure 3.**
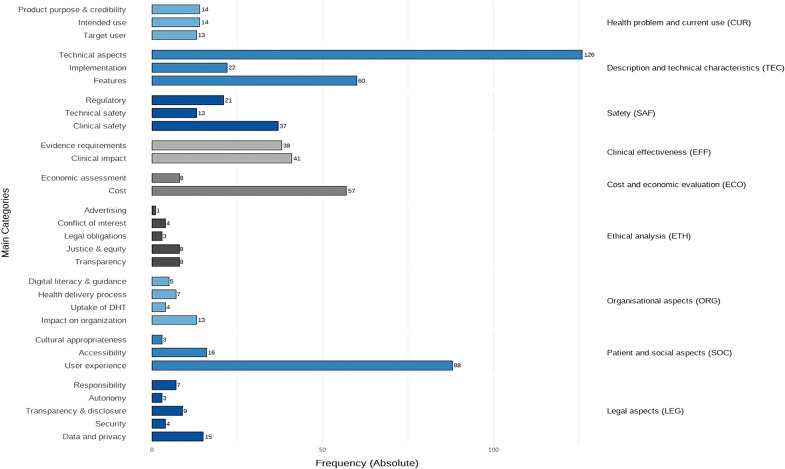
Frequencies (absolute) of main categories derived from the included frameworks.

The number of main categories per domain ranged from two to five. The CUR domain was assigned forty-one items. A total of 208 items were assigned to the TEC domain, making it one of the most frequently covered domains. The main category “Technical Aspects,” which contains the most assigned items, included subcategories such as data protection, data security and privacy, and interoperability. The main category “Features,” which also includes a significant number of items, encompassed subcategories like description and product information, function, and layout. The SAF domain comprised three main categories such as clinical safety, technical safety, and regulatory aspects, as some of the included frameworks incorporate regulatory compliance as part of the safety assessment. The EFF domain was assigned eighty-one items, while the ECO domain was assigned sixty-five items, with most items allocated to the main category “Cost.” In the ETH domain, twenty-four items were assigned, making it a less frequently covered domain in the frameworks. This is also the case for the ORG domain, which was assigned twenty-six items, and the LEG domain, where thirty-eight items were allocated. In contrast, 107 items were assigned to the SOC domain, making it one of the domains most frequently mentioned in the frameworks. The entire domain encompasses three main categories: “user experience,” “accessibility,” and “cultural appropriateness.” Figure 3 presents the frequency of these main categories across all nine HTA domains. The detailed frequency analysis of the HTA domains and associated items is provided in Supplementary File 4.

## Discussion

This scoping review provides a comprehensive overview of existing frameworks for assessing DHTs. Most frameworks focus on digital health applications in general and primarily aim to provide support and guidance for DHT assessment, with varying emphases on evaluation methods, data quality, and evidence requirements. Overall, a lifecycle approach is rarely considered, and classification schemes, which may necessitate different evidence requirements, are rare. Although evidence requirements are addressed more often, they typically remain broad and lack specificity.

The assessment domains covered by the included frameworks span health problem and current use, technical characteristics, safety, clinical effectiveness, economic aspects, ethics, organizational aspects, social aspects, and legal aspects, though their depth of coverage varies. While some frameworks align with the classical definition of an HTA framework, the inclusion criteria required only that at least one HTA Core Model domain needed to be addressed. As a result, the review also encompassed more narrowly focused frameworks that concentrate on a single domain, such as technical performance or regulatory compliance.

This review extracted 660 items from the frameworks, categorized into thirty-one main categories and forty-nine subcategories. The item count includes repetitions across frameworks and should not be interpreted as reflective of their overall importance or compared directly with the EUnetHTA Core Model, which comprises fewer items. Partial overlap existed between categories and subcategories, and some items could reasonably be assigned to multiple domains. For instance, the “Quality of information” item could be relevant to both the Effectiveness and Safety domains. Several domains are intertwined, which is a complexity also acknowledged in the EUnetHTA Core Model, which notes overlaps between domains such as organizational aspects, social aspects, and technical characteristics. This highlights the methodological challenge of domain classification in the context of HTA (9).

As outlined in the introduction, comprehensive frameworks for DHT evaluation are expected to cover a wide range of domains, including clinical, technical, economic, ethical, legal, social, and organizational aspects. However, our findings show that these domains are represented unevenly across existing frameworks. Certain domains, such as technical characteristics, were frequently and comprehensively addressed, whereas ethical and legal domains received comparatively limited attention. This imbalance mirrors previous findings showing that HTA reports tend to prioritize clinical effectiveness, economic evaluation, and technical features, while organizational or social aspects are frequently neglected (8;48). Such disparities may reflect perceived importance within HTA, highlighting the need for more attention to ELSI domains (49). Their underrepresentation risks overlooking critical considerations such as data privacy, security, and equitable access, which may compromise the safe, effective, and fair adoption of DHTs (50). While DHTs hold the potential to address disparities in health care, they may also pose a potential threat to equity in health care if they are not appropriately valued (51).

The review identified a variety of frameworks for assessing DHTs, aligning with findings from prior analyses which showed heterogeneity in structure, approach, and assessment elements (10). This diversity reflects the challenge of establishing a standard for evaluating DHTs. Overall, there is a lack of a clear definition of evidence criteria and requirements for evaluating DHTs.

The review emphasizes the need for a framework that can support harmonization efforts across countries and that includes the key issues and considerations for the assessment of DHTs that are currently included to varying degrees in the frameworks reviewed.

Finally, the dynamic nature of DHTs may necessitate ongoing evaluation and adaptation. Therefore, frameworks should be flexible and agile enough to address situations where a DHT has evolved in ways that render the initial assessment outdated, and to accommodate new features and types of digital technologies as well as the evolving evidence base. For instance, continuous monitoring and feedback mechanisms are essential elements to ensure that DHTs remain effective and safe over time (52).

This review did not distinguish between DHT-specific and general health technology assessment items, as many frameworks included both. The EUnetHTA Core Model was applied as a structured analytical framework to organize the extracted items across domains and to ensure comparability of content across the included DHT-specific frameworks. Future work could explore this distinction in more detail and consider structured alignment with the well-established EUnetHTA Core Model. Achieving greater harmonization across existing frameworks and reaching consensus on key evaluation criteria will be crucial to improving DHT assessments. It is important to consider the specific characteristics of DHTs and to develop tailored evaluation methods that meet the different requirements (53). A promising approach might be a modular framework that can be adapted depending on the area of application and technology.

### Limitations

Some limitations of this review should be acknowledged. First, time constraints in the search for relevant literature may have led to the exclusion of some relevant frameworks, particularly those published after the search period. Language restrictions may have had a similar effect. Some of the frameworks identified in this review date back to the early part of the specified time period and may no longer reflect current developments. Second, the use of specific keywords such as “framework” may have limited the search, although this was mitigated by a comprehensive hand search. Notably, many frameworks were identified through hand searching, which would not have been retrieved through database searches alone. However, some of the frameworks are accessible only on the websites of the HTA agencies. The analysis reflects the available evidence at the time of the literature search in a rapidly evolving field. Dynamic, web-based platforms and decision-support tools may therefore not be comprehensively identified. Overall, the use of numerous sources can also be considered a strength of this analysis. General HTA frameworks that include guidance on digital technologies but do not explicitly refer to DHTs were not captured by the search strategy, as the review focused on frameworks specifically developed for the assessment of DHTs. Finally, categorization of assessment items involved an element of subjectivity, an inherent limitation of qualitative synthesis (54). To address this, categorizations were cross-validated by at least one additional reviewer, and discussions within the team were conducted to ensure consistency and accuracy. While these limitations highlight areas for future improvement, the robustness of the review was strengthened through the use of diverse sources and rigorous methodological procedures.

## Conclusion

This scoping review identified a diverse array of frameworks for assessing DHTs, most of which focus on digital health applications and differ in their emphasis on evidence requirements. Lifecycle-based evaluation approaches are rarely incorporated, and only a few frameworks include classification methods for DHTs. The extent to which the EUnetHTA Core Model domains are addressed differs markedly across frameworks. The findings highlight the need for a comprehensive and standardized framework with explanations of the methodological approach to the different domains relevant to the assessment of DHTs. A potential approach to developing such a framework might involve a modular structure that allows adaptation based on the type of technology and the underlying features of the technology being assessed.

## Supporting information

10.1017/S0266462326103523.sm001 Rödiger et al. supplementary material Rödiger et al. supplementary material

## Supplementary material

The supplementary material for this article can be found at http://doi.org/10.1017/S0266462326103523.
