# Supplementary material for: Frameworks for assessing digital health technologies: a scoping review
Source: Int J Technol Assess Health Care. 2026 Mar 9;42(1):e24. doi: 10.1017/S0266462326103523 (PMC13071847; doi:10.1017/S0266462326103523)
Supplement: Rödiger et al. supplementary material [file S0266462326103523sup001.docx]

SUPPLEMENTARY FILES

## Supplementary 1 Searchstring

**PubMed**

| **#** |  | **Results (25 Apr 2024)** |
| --- | --- | --- |
| 1 | "telemedicine"[MeSH Terms] OR "digital technology"[MeSH Terms] OR "mobile applications"[MeSH Terms] OR "monitoring, ambulatory" [MeSH Terms] OR "digital health"[tiab] OR "digital therapeutic"[tiab] OR "digital health application*"[tiab] OR "DiHA"[tiab] OR "mobile health" [tiab] OR "mHealth"[tiab] OR "telehealth"[tiab] OR "telecare"[tiab] OR "web based intervention*"[tiab] OR "internet based intervention*"[tiab] OR "artificial intelligence"[tiab] OR "medical artificial intelligence"[tiab] OR "medical AI"[tiab] | 160,953 |
| 2 | (technology assessment, biomedical [MeSH Terms] OR "evaluat*"[tiab] OR "apprais*"[tiab] OR "appraisal"[tiab] OR "health technology assessment"[tiab] OR HTA [tiab] OR "technology assessment*"[tiab] OR "technology evaluation*"[tiab]) | 4,706,876 |
| 3 | ("framework*"[tiab] OR "guideline*" [tiab] OR "guidance*" [tiab]) | 1,081,521 |
| 4 | ("health" [MeSH Terms] OR "medicine" [MeSH Terms] OR "therapeutics" [MeSH Terms]) | 6,549,808 |
| ((1 AND (2 AND 3)) AND 4)  and 2015-2024 |  | **1,976** |

**Embase**

| **No.** | **Query** | **Results** | **Results Date** |
| --- | --- | --- | --- |
| #35. | #33 NOT #34 | 2,931 | 25 Apr 2024 |
| #34. | #33 AND 'conference abstract'/it | 1,283 | 25 Apr 2024 |
| #33. | #32 AND [2015-2024]/py | 4,214 | 25 Apr 2024 |
| #32. | #17 AND #23 AND #27 AND #31 | 4,872 | 25 Apr 2024 |
| #31. | #28 OR #29 OR #30 | 14,623,066 | 25 Apr 2024 |
| #30. | 'therapy'/exp | 11,118,470 | 25 Apr 2024 |
| #29. | 'medicine'/exp | 4,344,613 | 25 Apr 2024 |
| #28. | 'health'/exp | 945,962 | 25 Apr 2024 |
| #27. | #24 OR #25 OR #26 | 1,441,475 | 25 Apr 2024 |
| #26. | 'guidance*':ti,ab | 243,995 | 25 Apr 2024 |
| #25. | 'guideline*':ti,ab | 783,688 | 25 Apr 2024 |
| #24. | 'framework*':ti,ab | 473,657 | 25 Apr 2024 |
| #23. | #18 OR #19 OR #20 OR #21 OR #22 | 6,580,276 | 25 Apr 2024 |
| #22. | 'technology assessment*':ti,ab | 12,407 | 25 Apr 2024 |
| #21. | hta:ti,ab | 9,870 | 25 Apr 2024 |
| #20. | 'apprais*':ti,ab | 91,878 | 25 Apr 2024 |
| #19. | 'evaluat*':ti,ab | 6,498,514 | 25 Apr 2024 |
| #18. | 'biomedical technology assessment'/exp | 18,176 | 25 Apr 2024 |
| #17. | #1 OR #2 OR #3 OR #4 OR #5 OR #6 OR #7 OR #8 OR #9 OR #10 OR #11 OR #12 OR #13 OR #14 OR #15 OR #16 | 184,752 | 25 Apr 2024 |
| #16. | 'medical ai':ti,ab | 233 | 25 Apr 2024 |
| #15. | 'artificial intelligence':ti,ab | 49,173 | 25 Apr 2024 |
| #14. | 'internet based intervention*':ti,ab | 980 | 25 Apr 2024 |
| #13. | 'web based intervention*':ti,ab | 1,671 | 25 Apr 2024 |
| #12. | 'telecare':ti,ab | 1,024 | 25 Apr 2024 |
| #11. | 'telehealth':ti,ab | 16,865 | 25 Apr 2024 |
| #10. | 'mhealth':ti,ab | 6,990 | 25 Apr 2024 |
| #9. | 'mobile health':ti,ab | 7,465 | 25 Apr 2024 |
| #8. | diha:ti,ab | 64 | 25 Apr 2024 |
| #7. | 'digital health application*':ti,ab | 327 | 25 Apr 2024 |
| #6. | 'digital therapeutic':ti,ab | 347 | 25 Apr 2024 |
| #5. | 'digital health':ti,ab | 7,142 | 25 Apr 2024 |
| #4. | 'ambulatory monitoring'/exp | 12,359 | 25 Apr 2024 |
| #3. | 'mobile application'/exp | 28,033 | 25 Apr 2024 |
| #2. | 'digital technology'/exp | 5,754 | 25 Apr 2024 |
| #1. | 'telemedicine'/exp | 76,005 | 25 Apr 2024 |

## Supplementary 2 Included frameworks

| Study Abbreviation | Author (et al.) | Published Year | Title |
| --- | --- | --- | --- |
| Agarwal 2016 | Agarwal, S. et al. | 2016 | Guidelines for reporting of health interventions using mobile phones: mobile health (mHealth) evidence reporting and assessment (mERA) checklist |
| Agency for Healthcare Research and Quality 2022 | Agency for Healthcare Research and Quality | 2022 | Evaluation of Mental Health Mobile Applications |
| American Psychiatric Association 2019 | American Psychiatric Association | 2019 | App Evaluation Model |
| Anxiety and Depression Association of America 2022 | Anxiety and Depression Association of America | 2022 | Mental Health Apps Assessment Model |
| AQuAS 2023 | Segur-Ferrer, J. et al. | 2023 | Health Technology Assessment Framework: Adaptation for Digital Health Technology Assessment. |
| Baumel 2017 | Baumel, A. et al. | 2017 | Enlight: A Comprehensive Quality and Therapeutic Potential Evaluation Tool for Mobile and Web-Based eHealth Interventions |
| Bertl 2023 | Bertl, M. et al. | 2023 | Systematic AI Support for Decision-Making in the Healthcare Sector: Obstacles and Success Factors |
| Betton 2017 | Betton, V et al. | 2017 | Framework for the effectiveness evaluation of mobile (mental) health tools |
| BfArM 2020 | Bundesinstitut für Arzneimittel und Medizinprodukte (BfArM) | 2020 | The Fast-Track Process for Digital Health Applications (DiGA) according to Section 139e SGB V A Guide for Manufacturers, Service Providers and Users |
| Biswas 2021 | Biswas, M. et al. | 2021 | ACCU3RATE: A mobile health application rating scale based on user reviews |
| Bongiovanni-Delaroziére 2017 | Bongiovanni-Delaroziére, I. et al. | 2017 | Economic evaluation methods applied to telemedicine: From a literature review to a standardized framework |
| British Standards Institution 2015 | British Standards Institution | 2015 | Health and wellness apps “Quality criteria across the life cycle“ Code of practice |
| Camacho 2020 | Camacho, E. et al. | 2020 | Technology Evaluation and Assessment Criteria for Health Apps (TEACH-Apps): Pilot Study. |
| Chan 2015 | Chan, S. et al. | 2015 | Towards a Framework for Evaluating Mobile Mental Health Apps |
| Coravos 2020 | Coravos, A. et al. | 2020 | Modernizing and designing evaluation frameworks for connected sensor technologies in medicine |
| Dawson 2020 | Dawson, R.M. et al. | 2020 | What makes a good health 'app'? Identifying the strengths and limitations of existing mobile application evaluation tools |
| Defense Health Agency 2018 | Defense Health Agency | 2018 | U.S. Department of Defense Mobile Health Practice Guide |
| Digital Health Assessment Framework, ORCHA, 2022 | ORCHA | 2022 | Digital Health Assessment Framework, ORCHA |
| Digital Therapeutics Alliance 2022 | Digital Therapeutics Alliance | 2022 | DTx Value Assessment & Integration Guide |
| Dulude 2023 | Dulude, C. et al. | 2023 | A pediatric virtual care evaluation framework and its evolution using consensus methods |
| Haute Autorité de Santé 2016 | Haute Autorité de Santé | 2016 | Good Practice Guidelines on Health Apps and Smart Devices (Mobile Health or mHealth) |
| Haverinen 2019 | Haverinen, J. et al. | 2019 | Digi-HTA: Health technology assessment framework for digital healthcare services |
| Henson 2019 | Henson, P. et al. | 2019 | Deriving a practical framework for the evaluation of health apps |
| Hussain 2021 | Hussain, MS.et al | 2021 | Technology assessment framework for precision health applications. |
| ISO 2021 | ISO | 2021 | ISO/TS 82304-2:2021(en) Health software ”Part 2: Health and wellness apps” Quality and reliability |
| KNMG 2016 | KNMG | 2016 | Medical App Checker: Evaluation of Mobile Medical Apps |
| Kowatsch 2019 | Kowatsch, T. et al. | 2019 | A design and evaluation framework for digital health interventions |
| Lagan 2021a | Lagan, S. et al. | 2021a | Evaluating evaluation frameworks: A scoping review of frameworks for assessing health apps |
| Lagan 2021b | Lagan, S. et al. | 2021b | Mental Health App Evaluation: Updating the American Psychiatric Association's Framework Through a Stakeholder-Engaged Workshop. |
| Lantzsch 2022 | Lantzsch, H. et al. | 2022 | Benefit Assessment and Reimbursement of Digital Health Applications: Concepts for Setting Up a New System for Public Coverage |
| Leigh 2017 | Leigh, S. et al. | 2017 | Effective? Engaging? Secure? Applying the ORCHA-24 framework to evaluate apps for chronic insomnia disorder |
| Levine 2020 | Levine, D. et al. | 2020 | Design and testing of a mobile health application rating tool |
| Mackey 2022 | Mackey, R. et al. | 2022 | A Novel Method for Evaluating Mobile Apps (App Rating Inventory): Development Study |
| Mathews 2019 | Mathews, S. et al. | 2019 | Digital health: a path to validation |
| McMillan 2016 | McMillan, B. et al. | 2016 | Quality assessment of a sample of mobile app-based health behavior change interventions using a tool based on the National Institute of Health and Care Excellence behavior change guidance |
| Mental Health Commission 2018 | Mental Health Commission | 2018 | Mental Health Apps: How to Make an Informed Choice |
| mhealth Belgium 2024 | mhealth Belgium | 2024 | mHealthBELGIUM Validation pyramid |
| Moshi 2020 | Moshi MR. et al. | 2020 | Development of a health technology assessment module for evaluating mobile medical applications. |
| Murray 2016 | Murray, E. et al. | 2016 | Evaluating Digital Health Interventions |
| National Institute for Health and Care Excellence 2022 | National Institute for Health and Care Excellence | 2022 | Evidence standards framework (ESF) for digital health technologies |
| National Library of Medicine 2022 | National Library of Medicine | 2022 | Evaluating Mobile Apps |
| Nebeker 2020 | Nebeker, C. et al. | 2020 | Development of a decision-making checklist tool to support technology selection in digital health research |
| NHS Digital 2019 | NHS Digital | 2019 | Digital Assessment Questions V2.2 |
| NHS England 2021 | NHS England | 2021 | Digital Technology Assessment Criteria (DTAC) |
| NordDEC 2024 | NordDEC | 2024 | NORDIC DIGITAL HEALTH EVALUATION CRITERIA ONE ASSESSMENT TO MEET THE DIGITAL HEALTH EVALUATION REQUIREMENTS IN DENMARK, FINLAND, ICELAND, NORWAY, AND SWEDEN. |
| Nouri 2018 | Nouri, R. et al. | 2018 | Criteria for assessing the quality of mHealth apps: a systematic review |
| O'Rourke 2020 | O'Rourke, T. et al. | 2020 | Development of a Multidimensional App-Quality Assessment Tool for Health-Related Apps (AQUA) |
| Pearson 2023 | Pearson, SD. et al. | 2023 | Institute for Clinical and Economic Revie, Peterson Health Technology Institute value assessment framework for digital health technologies |
| Quintana 2020 | Quintana, Y. & Torous, J. | 2020 | A Framework for Evaluation of Mobile Apps for Youth Mental Health |
| Roberts 2021 | Roberts, AE. Et al. | 2021 | Evaluating the quality and safety of health-related apps and e-tools: Adapting the Mobile App Rating Scale and developing a quality assurance protocol |
| Silberman 2023 | Silberman, J. et al. | 2023 | Rigorous and rapid evidence assessment in digital health with the evidence DEFINED framework |
| Stoyanov 2015 | Stoyanov, SR. et al. | 2015 | Mobile App Rating Scale: A New Tool for Assessing the Quality of Health Mobile Apps |
| Tarricone 2022 | Tarricone R. et al. | 2022 | Recommendations for developing a lifecycle, multidimensional assessment framework for mobile medical apps. |
| Vokinger 2020 | Vokinger, KN. et al. | 2020 | Digital health and the COVID-19 epidemic: an assessment framework for apps from an epidemiological and legal perspective |
| Wagneur 2022 | Wagneur, N. et al. | 2022 | Assessing a New Prescreening Score for the Simplified Evaluation of the Clinical Quality and Relevance of eHealth Apps: Instrument Validation Study |
| Wu 2021 | Wu, K-L. et al. | 2021 | Characteristics and Quality of Mobile Apps Containing Prenatal Genetic Testing Information: Systematic App Store Search and Assessment |
| Wyatt 2015 | Wyatt, J. et al. | 2015 | What makes a good clinical app? Introducing the RCP Health Informatics Unit checklist |
| Wykes 2019 | Wykes, T. & Schueller, S. | 2019 | Why Reviewing Apps Is Not Enough: Transparency for Trust (T4T) Principles of Responsible Health App Marketplaces |
| Xcertia 2019 | Xcertia | 2019 | Xcertia mHealth App Guidelines |
| Zelmer 2018 | Zelmer, J. et al. | 2018 | An Assessment Framework for e-Mental Health Apps in Canada: Results of a Modified Delphi Process |

## Supplementary 3 Summary of the coverage of the HTA domains for each framework

| **Framework** | **Health problem and current use of technology (CUR)** | **Description and technical characteristics of technology (TEC)** | **Safety (SAF)** | **Clinical effectiveness (EFF)** | **Cost and economic evaluation (ECO)** | **Ethical analysis (ETH)** | **Organisational aspects (ORG)** | **Patients and Social aspects (SOC)** | **Legal aspects (LEG)** |
| --- | --- | --- | --- | --- | --- | --- | --- | --- | --- |
| Agarwal 2016 |  | x | x |  | x |  |  | x |  |
| Agency for Healthcare Research and Quality 2022 |  | x | x | x | x |  |  | x | x |
| American Psychiatric Association 2019 |  | x | x | x | x | x |  | x | x |
| Anxiety and Depression Association of America 2022 |  | x |  | x |  |  |  | x |  |
| AQuAS 2023 | X | x | x | x | x | x | x | x | x |
| Baumel 2017 |  | x |  | x |  |  |  | x |  |
| Bertl 2023 |  | x |  |  |  |  |  |  |  |
| Betton 2017 | X | x |  | x | x |  |  |  |  |
| BfArM 2020 |  | x | x | x |  | x | x |  |  |
| Biswas 2021 |  | x |  |  |  |  |  | x |  |
| Bongiovanni-Delaroziére 2017 | X |  |  |  | x |  | x | x |  |
| British Standards Institution 2015 |  | x |  |  |  |  |  |  |  |
| Camacho 2020 |  | x |  |  | x |  |  |  |  |
| Chan 2015 |  | x |  | x |  |  |  | x |  |
| Coravos 2020 |  | x | x | x | x |  |  | x |  |
| Dawson 2020 |  |  |  | x |  |  |  | x |  |
| Defense Health Agency 2018 |  | x |  |  |  |  |  | x |  |
| Digital Health Assessment Framework, ORCHA, 2022 |  | x |  |  |  |  |  | x |  |
| Digital Therapeutics Alliance 2022 |  | x |  | x | x |  |  | x |  |
| Dulude 2023 | X |  |  | x |  |  |  |  |  |
| Haute Autorité de Santé 2016 |  | x | x | x | x |  | x | x | x |
| Haverinen 2019 | X | x | x | x | x |  |  | x |  |
| Henson 2019 |  | x |  | x | x |  |  | x | x |
| Hussain 2021 |  | x |  | x |  |  |  | x |  |
| ISO 2021 | x | x | x | x |  | x |  | x |  |
| KNMG 2016 |  | x | x | x |  |  |  | x |  |
| Kowatsch 2019 |  | x | x | x |  | x |  | x |  |
| Lagan 2021a |  | x |  |  |  |  |  | x |  |
| Lagan 2021b |  | x |  | x | x |  |  |  |  |
| Lantzsch 2022 |  |  |  | x |  |  |  |  |  |
| Leigh 2017 |  | x |  | x |  |  |  | x |  |
| Levine 2020 |  | x | x |  | x |  | x | x |  |
| Mackey 2022 |  | x |  | x |  |  |  | x |  |
| Mathews 2019 |  | x |  | x | x |  | x | x |  |
| McMillan 2016 | x | x | x | x |  |  |  | x |  |
| Mental Health Commission 2018 | x | x | x | x | x | x |  | x | x |
| mHealthBELGIUM | x | x | x | x | x |  | x |  |  |
| Moshi 2020 | x | x |  | x | x | x | x | x | x |
| Murray 2016 | x | x | x | x | x |  | x | x |  |
| National Institute for Health and Care Excellence 2022 | x | x | x | x | x | x | x | x |  |
| National Library of Medicine 2022 |  | x |  | x | x | x |  | x |  |
| Nebeker 2020 |  | x | x | x |  | x |  | x |  |
| NHS Digital 2019 |  | x | x | x | x |  |  | x |  |
| NHS England 2021 |  | x | x |  |  |  |  | x |  |
| NordDEC 2024 |  | x | x |  |  |  |  | x | x |
| Nouri 2018 |  | x | x |  |  |  |  | x | x |
| O'Rourke 2020 | x | x |  | x | x |  |  | x | x |
| Pearson 2023 |  |  | x | x | x |  |  |  |  |
| Quintana 2020 | x | x |  | x | x | x |  | x | x |
| Roberts 2021 | x | x |  |  |  |  |  | x |  |
| Silberman 2023^^[[1]](#footnote-1)^^ |  |  |  |  |  |  |  |  |  |
| Stoyanov 2015 |  | x |  | x | x |  |  | x |  |
| Tarricone 2022 |  |  |  | x | x | x | x | x |  |
| Vokinger 2020 | x | x | x | x | x | x |  | x | x |
| Wagneur 2022 | x | x |  |  | x |  | x | x |  |
| Wu 2021 |  | x | x | x | x |  |  |  |  |
| Wyatt 2015 | x | x | x | x | x |  |  |  |  |
| Wykes 2019 | x | x |  | x |  |  |  |  |  |
| Xcertia 2019 | x | x | x |  |  | x |  | x | x |
| Zelmer 2018 | x | x |  | x | x | x |  | x | x |

Note: x=Coverage

## Supplementary 4 Frequency analysis of the HTA domains and associated items, derived from included frameworks

| HTA Domain | Main | Subcategory (if available) | No. of frameworks the categorisation is based on | No. of items assigned to domain |
| --- | --- | --- | --- | --- |
| **Health problem and current use (CUR)** | Target user |  |  | 13 |
|  | Intended use |  |  | 14 |
|  | Product purpose & credibility |  |  | 14 |
| **Total** |  |  | **21** | **41** |
| **Description and technical characteristics (TEC)** | Features | Purpose |  | 11 |
|  |  | Function |  | 16 |
|  |  | Performance |  | 2 |
|  |  | Description & product information |  | 18 |
|  |  | Layout |  | 13 |
|  | Implementation | Access control |  | 2 |
|  |  | Infrastructure |  | 14 |
|  |  | User engagement |  | 6 |
|  | Technical aspects | Data protection |  | 10 |
|  |  | Data security & privacy |  | 50 |
|  |  | Interoperability, Connectivity & Data sharing |  | 22 |
|  |  | Transparency & Control |  | 7 |
|  |  | Technical stability & service |  | 6 |
|  |  | Updates & maintenance |  | 14 |
|  |  | Data management |  | 17 |
| **Total** |  |  | **45** | **208** |
| **Safety (SAF)** | Clinical safety | Harm consideration |  | 14 |
|  |  | Risk assessment |  | 10 |
|  |  | Professional assurance |  | 5 |
|  |  | Risk preventive measures |  | 3 |
|  |  | Company‘s measures |  | 5 |
|  | Technical safety | User protection |  | 7 |
|  |  | Quality of information |  | 6 |
|  | Regulatory aspects | Compliance with requirements |  | 11 |
|  |  | Documentation |  | 6 |
|  |  | 3rd-party connection |  | 1 |
|  |  | Recall system |  | 1 |
|  |  | Non-medical device declaration |  | 2 |
| **Total** |  |  | **27** | **71** |
| **Clinical Effectiveness (EFF)** | Clinical impact | Medical focus |  | 10 |
|  |  | Content quality |  | 16 |
|  |  | Clinical benefit |  | 15 |
|  | Evidence requirements | Evidence base |  | 28 |
|  |  | Validity |  | 10 |
|  |  | Comparator |  | 3 |
| **Total** |  |  | **43** | **81** |
| **Cost and economic evaluation (ECO)** | Cost | Business model |  | 8 |
|  |  | Cost to organization |  | 10 |
|  |  | Cost to users (direct and indirect) |  | 5 |
|  |  | Price of DHT |  | 4 |
|  |  | Transparency |  | 20 |
|  |  | Reimbursement |  | 10 |
|  | Economic assessment | Economic benefit |  | 6 |
|  |  | Economic evaluation |  | 2 |
| **Total** |  |  | **32** | **65** |
| **Ethical analysis (ETH)** | Transparency |  |  | 8 |
|  | Justice & Equity |  |  | 8 |
|  | Legal obligations |  |  | 3 |
|  | Conflict of interest |  |  | 4 |
|  | Advertising |  |  | 1 |
| **Total** |  |  | **15** | **24** |
| **Organisational aspects (ORG)** | Impact on organization |  |  | 13 |
|  | Uptake of DHT |  |  | 4 |
|  | Health delivery process |  |  | 7 |
|  | Digital literacy & Guidance |  |  | 5 |
| **Total** |  |  | **12** | **29** |
| **Patient and social aspects (SOC)** | User experience | Usability |  | 32 |
|  |  | Ease of Use |  | 12 |
|  |  | User engagement |  | 9 |
|  |  | User inclusion |  | 11 |
|  |  | User feedback |  | 18 |
|  |  | Patient empowerment |  | 2 |
|  |  | Social aspects |  | 2 |
|  |  | Support |  | 2 |
|  | Accessibility |  |  | 16 |
|  | Cultural appropriateness |  |  | 3 |
| **Total** |  |  | **46** | **107** |
| **Legal aspects (LEG)** | Data and Privacy |  |  | 15 |
|  | Security |  |  | 4 |
|  | Transparency & Disclosure |  |  | 9 |
|  | Autonomy |  |  | 3 |
|  | Responsibility |  |  | 7 |
| **Total** |  |  | **14** | **38** |

Note: CUR=Health Problem and Current Use; TEC=Description and technical characteristics; SAF=Safety; EFF=Clinical Effectiveness; ECO=Cost and economic evaluation; ETH=Ethical analysis (ETH); ORG=Organisational aspects; SOC=Patient and social aspects

1. Framework of Silbermann et al. does not contain any crosses, as it could not be assigned to any HTA domains after review. However, it was included as a framework because it contains the “Checklist of Evidence Quality Criteria for Digital Health Interventions” and data was extracted for “evidence requirements”. [↑](#footnote-ref-1)
